# Saunders’ American Year-Book of Medicine and Surgery

**Published:** 1896-01

**Authors:** 


SAUNDERS' AMERICAN YEAR-BOOK OF MEDICINE AND SURGERY. Edited by George M. Gould, A. M., M. D., assisted by Eminent American Physicians and Teachers.

The popular publisher, W. B. Saunders, of 925 Walnut St., Philadelphia, will issue the first edition of his American Year-Book of Medicine and Surgery early in January, 1896. This book will be edited by Dr. George M. Gould, assisted by a corps of eminent American physicians and teachers. It is the purpose of the editors to search out in medical periodicals that which is of most practical value to the physician, and place before them in convenient form an epitomization of this literature. The following is a portion of Mr. Saunders' announcement of this book:

"It is the special purpose of the editor, whose experience peculiarly qualifies him for the preparation of this work, not only to review the contributions to American journals, but also the methods and discoveries reported in the leading medical journals of Europe, thus enlarging the survey and making the work characteristically international. These reviews will not simply be a series of undigested abstracts, indiscriminately run together, nor will they be retrospective of "news" *one or two years old*, but the treatment presented will be *synthetic* and *dogmatic*, and will include only what is new. Moreover, through expert condensasation by experienced writers, these discussions will be comprised in a single volume.

The work will be replete with original and selected illustrations skillfully reproduced, for the most part, in Mr. Saunders' own studios established for the purpose, thus insuring accuracy in delineation, affording efficient aids to a right comprehension of the text, and adding to the attractiveness of the volume."

